# Beliefs, Attitudes and Self-Efficacy of Australian Veterinary Students Regarding One Health and Zoonosis Management

**DOI:** 10.3390/ani9080544

**Published:** 2019-08-10

**Authors:** Ihab Habib, Wing Sze Lam, Hamid Reza Sodagari, Peter Irons, Mieghan Bruce

**Affiliations:** 1 School of Veterinary Medicine, College of Science, Health, Education and Engineering, Murdoch University, Perth 6150, Australia; 2 Veterinary Medicine Department, College of Food and Agriculture, United Arab of Emirates University (UAEU), Al Ain P.O. Box 15551, UAE

**Keywords:** Australia, One Health, veterinary public health, preventive veterinary medicine, interprofessional collaboration

## Abstract

**Simple Summary:**

The One Health approach provides a model for educating students, trainees and professionals in a systems approach to providing improved patient care and promoting healthy environments that benefit all species. In the present study, we designed an online survey that was communicated to senior veterinary students in a number of Australian veterinary schools. The survey evaluated the willingness of future veterinary graduates to participate positively in zoonoses (diseases transmitted between animals and humans) management through the adoption of the One Health approach in their future career. All respondents were willing to assist physicians in the management of zoonotic cases involving both patients and clients. The veterinary students were equally concerned about ensuring the best care for both animals (patients) and humans (clients). Our results demonstrate that there is strong support for future Australian veterinarians in the field of One Health.

**Abstract:**

This study aimed to explore the beliefs and attitudes of a group of senior veterinary students regarding One Health and to evaluate their levels of confidence in advising the general public on preventative health issues at the human–animal interface. An online survey was communicated to senior veterinary students who were in their last two years of study. The questionnaire covered beliefs and attitudes, issues concerning the animal–human interface and participants’ confidence in diagnosing zoonoses. In total, 175 students from five Australian veterinary schools/colleges completed the online survey. The majority (96%) of students considered it their duty to promote the One Health approach, but only 36% believed there were sufficient practical frameworks for Australian veterinarian graduates to promote One Health. Interestingly, 81% (142/175) of respondents believed that veterinarians were more knowledgeable than physicians in managing zoonotic cases. Of the final-year students (n = 77), only 39% and 36% were confident in their ability to diagnose zoonoses in common companion animals and production animals, respectively. However, the number of those confident to diagnose zoonoses transmitted from wildlife was notably lower (22% (17/77)). Next-generation Australian veterinarians are keen to embrace their role in interprofessional collaboration; however, training efforts are required to reassure future veterinarians on aspects of zoonoses and One Health.

## 1. Introduction

The One Health approach recognises the health connections between humans and animals and their shared environments. It promotes professional cooperation between physicians, veterinarians and others to address complex problems affecting multiple species and pathogens in changing environments [1]. The One Health approach is particularly relevant to the control of emerging and re-emerging infectious diseases and zoonoses and combatting antimicrobial resistance. The One Health approach is also applicable to noninfectious diseases and broader issues such as food safety, food security and sustainable biosecurity [2,3].

Over the past decade in Australia, the One Health approach has rapidly gained recognition as an efficacious and expeditious approach to addressing some of today’s most complex problems [4]. Australia’s rich flora and fauna inhabit various climatic zones that vary from high alpine to Mediterranean to wet tropical. The richness and diversity of Australia’s ecological zones along with its diverse agricultural industries and rapid changes in land use have been associated with the emergence of zoonotic diseases. Australia has experienced emerging infectious disease events in the past decade, notably Australian bat lyssavirus and Hendra virus. In response to these events, a multisectoral One Health approach involving virological, ecological and biomedical teams was critical in managing the emergence of these viruses [5,6]. Along with emerging infectious diseases, the emergence of antimicrobial resistance is another issue for which the One Health approach is highly relevant. The National Antimicrobial Resistance Strategy 2015–2019 was released by the Australian government to guide the response to the threat of antibiotic misuse and resistance [7]. This strategy calls for a nationally coordinated One Health surveillance of antimicrobial usage and resistance and the minimisation of the development of resistance in livestock and companion animals as essential for addressing the emerging issue of antimicrobial resistance.

Recognising the value of the One Health approach and the need for training future One Health task forces, Australian universities have started incorporating the topic of One Health in their curricula. In total, eight formal academic offerings and one standalone training workshop in One Health and EcoHealth were identified in Australian universities [8]. The majority of courses that provide credit towards a degree are offered in undergraduate and postgraduate degrees in veterinary schools. The Australian veterinary medical profession is poised to become actively engaged in the One Health approach. However, educating the next generation of One Health veterinarians still faces challenges. Although several veterinary schools offer undergraduate and postgraduate training opportunities, the time allocated and the credit load for One Health courses and units varies widely between universities. Additionally, the training activities identified in Australian universities are based on different curricula and pedagogies depending on the disciplinary focus of the lead academic unit. Aside from degrees in veterinary schools, there was only one bachelor’s degree program and one intensive course in health faculties offering academic units in One Health [8]. Given that training is largely provided within restricted disciplinary boundaries, this presents a challenge for the development of One Health professionals.

Little is currently known about Australian veterinary students’ perceptions of the One Health approach, including their readiness and motivation to take part in practising this approach in their future careers. Hence, the aim of this study was to conduct a pilot assessment of veterinary students’ beliefs and attitudes regarding their potential contributions to One Health and to evaluate their level of confidence in advising the general public on preventative health issues at the human–animal interface.

## 2. Material and Methods

### 2.1. Survey Setting

Seven universities in Australia offer veterinary degrees, which involve between five and seven years of study depending on the university. The survey was open to all senior veterinary students in their final two years of education. Using the Survey Monkey platform, we developed a 21-question/opinion statement survey (see Table 1). We gathered basic demographic information and provided a series of statements regarding students’ beliefs and attitudes towards One Health and their perspectives on interprofessional collaboration at the animal–human interface. Responses to statements were based on a 5-point Likert scale [9] (strongly agree, somewhat agree, neither agree nor disagree, somewhat disagree and strongly disagree). A series of statements was also presented to evaluate respondents’ self-efficacy in advising the general public regarding preventative health issues and diagnosis of zoonoses. The survey questions on self-efficacy in diagnosis of zoonoses was only directed toward final-year students, as such topics are usually dealt with in the last couple of years (clinical tracks) of the veterinary curriculum; students in their earlier study years will not have enough training exposure in such clinical diagnostic aspects. Responses related to confidence levels were based on a 5-point Likert scale (confident, somewhat confident, neither confident nor not confident, somewhat not confident and not at all confident). For each quantitative survey item, the option to provide open-ended qualitative feedback (if students wished to expand on or justify their response) was provided via a comment box. The survey was developed with two veterinary public health and epidemiology consultants and piloted by five veterinary students prior to distribution. The survey was available online for a period of six months (February–August 2018). We computed simple descriptive statistics (frequencies and percentages) for each survey question using tools embedded in the Survey Monkey platform.

### 2.2. Respondent Recruitment and Survey Distribution

We recruited respondents indirectly through their academic mentors. Briefly, project information and invitation packages were emailed to veterinary public health and epidemiology academics across seven Australian universities. Peer academics were requested to disseminate the survey to the target veterinary student population at their home institution (note that students were not contacted directly by our researchers). Utilising the university administrators’ mailing lists, the network of academics sent emails, which included a brief introduction to the survey and a link to access the survey, to senior veterinary students at each school. Two reminder emails were sent, two months after the survey opened and two weeks before it closed, respectively. In total, academic representatives of five (out of seven) veterinary schools responded and confirmed that they had disseminated the survey to senior veterinary students (students in their last two years, n = 800) at their local institutions.

### 2.3. Ethics

This study was approved by the Murdoch University Human Research Ethics Committee (Approval 2017/266). Each respondent provided informed consent prior to commencing the online questionnaire. The aims and objectives of the study were explained to all respondents and the confidentiality of their information was confirmed.

## 3. Results

Of the 800 students emailed, 175 responses from senior veterinary students at five Australian universities were received (Table 1), representing an overall response rate of 21.8%. Demographic information is outlined in Table 2. Of the 175 respondents, 119 (68%) were female. Around half of the respondents (52%) were studying at Murdoch University in Western Australia, while the other half were enrolled in universities located in the eastern states of Australia.

**Figure 1:**
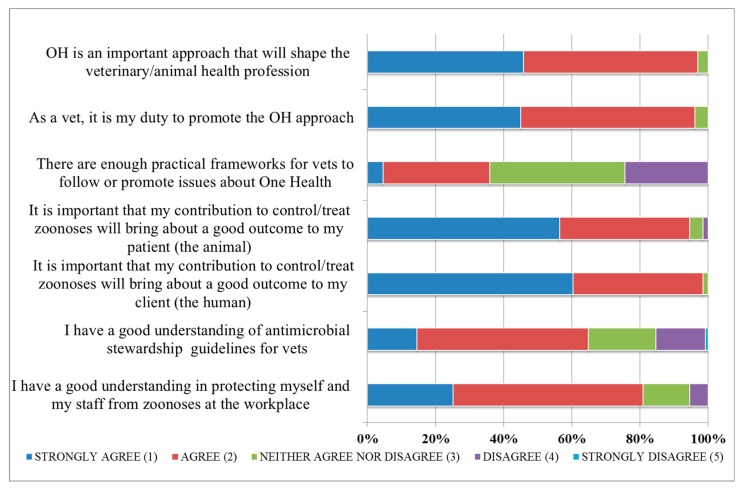
Perceived beliefs and attitudes expressed by 175 senior veterinary student respondents.

Of the students surveyed, the majority (97%) agreed/strongly agreed that One Health is an important approach that will shape the veterinary profession, and 96% expressed that it was their duty to promote such an approach in their future careers. However, only 36% agreed/strongly agreed that there are enough practical frameworks for veterinarians to promote One Health in Australia. In the follow-up questioning, 97% and 98% of respondents agreed/strongly agreed that it is important that their contribution to the management of zoonoses adds value to the health of animals and humans, respectively. Many respondents perceived that they had a good understanding of antimicrobial stewardship (65% agreeing or strongly agreeing) and personal biosecurity in the workplace (81%) (Figure 1).

**Figure 2:**
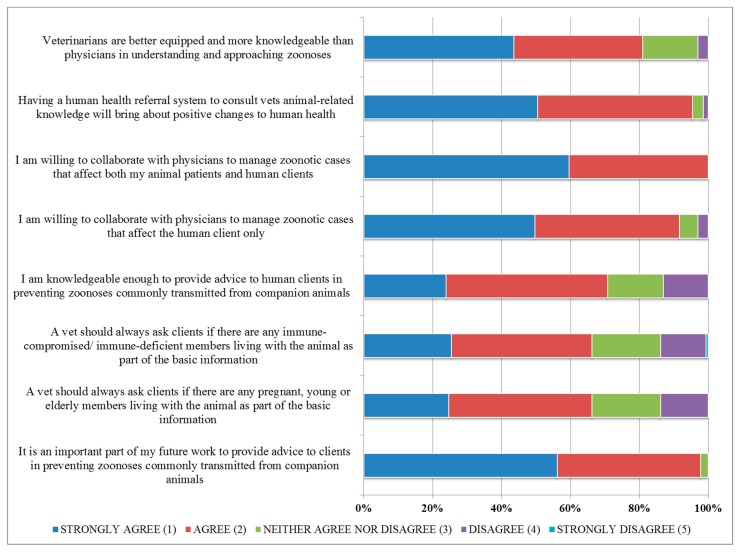
Perceived opinions expressed by 175 senior veterinary student respondents on interprofessional collaboration at the animal–human interface.

All respondents (100%) expressed their willingness (agreeing or strongly agreeing) to collaborate with physicians in managing cases of zoonoses at the animal patient–human client interface. Interestingly, 95% of students agreed/strongly agreed that a health referral system that involves consulting veterinarians on animal–human-related matters would be a positive change in the current health system. This was not surprising given that 81% of the respondents agreed/strongly agreed that veterinarians are more knowledgeable than physicians in approaching zoonoses and 71% believed they were sufficiently knowledgeable to provide preventative health advice to human clients on zoonosis prevention (Figure 2).

**Figure 3:**
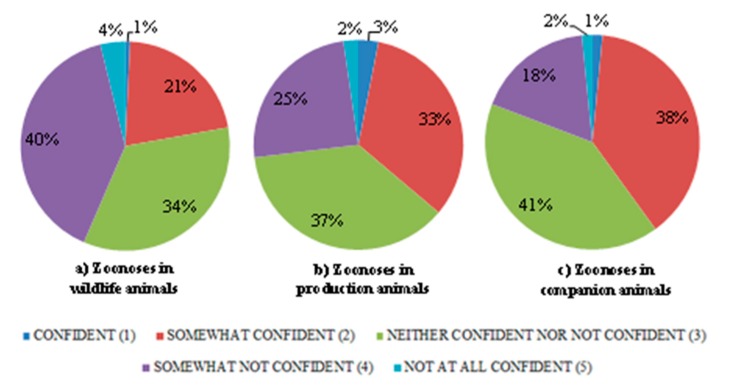
Responses of final-year veterinary student (n = 77) survey respondents to statements on self-efficacy in diagnosing zoonoses in different animal groups: (**a**) Wildlife animals, (**b**) Production animals, (**c**) Companion animals.

The self-efficacy of the final-year students (n = 77) surveyed regarding their level of confidence in diagnosing zoonoses varied in relation to animal groups (Figure 3). Of the students surveyed, 22% felt confident/somewhat confident in diagnosing zoonoses in wildlife. On the other hand, 39% of students expressed confidence in diagnosing zoonoses in companion animals, and 36% of them were confident in diagnosing zoonoses in production animals (Figure 3).

## 4. Discussion

The veterinary medicine profession routinely operates at the interface between animals, humans and the environment [1,2]. How veterinary graduates perceive that the role of the One Health approach in their future careers may have a significant impact on the overall resilience of preventative health care systems worldwide [10]. The results of the present survey show that Australian veterinary students have a positive perspective on the One Health approach and believe in its ability to improve both animal and human health. The survey found that a large majority (96%) of the students considered it their duty to promote the One Health approach. The positive reception towards the topic highlighted in the present survey reflects that the undergraduate curriculum provides a natural venue to prepare future veterinarians for applying the One Health approach after they graduate. One Health is a relatively new topic in Australian universities and has been delivered mainly through the efforts of several veterinary schools in recent years [8]. Veterinary medicine education in Australia provides a positive model at both the academic and professional level to actively go beyond its professionally protected comfort zone of licensing to build operational collaboration with other public health disciplines and sectors involved in the development of One Health at both national and international levels.

In the present survey, only 36% of students believed there were sufficient practical frameworks for newly graduating Australian veterinarians to promote the One Health approach. Similar to many countries, there is an obvious gap in integration and collaboration across the human health, animal health and ecology/wildlife sectors in Australia [11,12]. This has also been observed in a global review of One Health research [13]. It is clear that in major government departments (e.g., Department of Health and Department of Agriculture), there is limited systematic engagement between wildlife, medical and veterinary scientists and no apparent engagement with social scientists, economists and environmental health scientists [8,14]. The major action plans for One Health frameworks in Australia are responsive (ad hoc) in nature, similar to what has been observed in the management of several emerging infectious diseases (e.g., Hendra virus and Q fever) in the past two decades [13,14]. To support the formal role of Australian veterinarians in joint disease surveillance efforts, which has been shown to be extremely useful in the tracking of zoonoses, antimicrobial resistance and population-based surveillance of foodborne pathogens should be specifically explored.

The results of the present survey suggest that students enrolled in veterinary schools in Australia are mostly willing to collaborate with physicians to prevent and manage zoonotic cases. It is worth highlighting that students were equally concerned about ensuring the best care for both animals (patients) and humans (clients). These results are positive and affirm the crucial leadership role of veterinarians in the control of zoonotic diseases ahead of spillover to human populations. Interestingly, 81% (142/175) of respondents believed that veterinarians are more knowledgeable than physicians in approaching zoonotic cases. Given that veterinarians have multispecies training and are more familiar than physicians with management strategies involving more than one species, this view may be a natural outcome of the education model. Nevertheless, previous research exploring zoonosis prevention in Europe and the United States has indicated that physicians also believe that veterinarians are more knowledgeable about zoonoses than are members of their own profession [15,16]. In the individual health setting, collaborative input from both veterinarians and physicians would help in assessing a patient’s potential zoonotic disease risk from animal exposure [17,18]. These collaborative efforts would increase our understanding of how zoonoses expand their host range and, ultimately, would improve prevention and control strategies.

The surveyed veterinary students indicated variable levels of confidence depending on the category of animals regarding their perceived ability to diagnose common zoonotic diseases. The highest level of confidence was associated with zoonoses in companion animals, followed by production animals, and a notably lower level of confidence concerning zoonoses transmitted from wildlife (22%) (Figure 3). These differences may be attributable to the level of training delivered in the respective topics. For example, in the fourth year of Murdoch University’s applied veterinary medicine course, clinical skills are taught in units categorised by animal species. Units on companion animals and production animals (bovine, ruminant, porcine and poultry) carry 9 credit points each and the equine unit carries 4 credit points. However, the unit on wildlife, which includes avian and exotic pets, carries only 2 credit points [19]. University credits are in proportion to the amount of contact time spent in units.

Throughout history, both in Australia and worldwide, wildlife has been an important source of infectious diseases transmissible to humans. Zoonoses with a wildlife reservoir have a wide spectrum of transmission modes and several zoonotic agents may be transmitted directly from wildlife to humans [20]. For example, *Francisella tularensis*, the causative agent of tularaemia, can be transmitted by skin contact with an infested, diseased or dead hare or rodent; the rabies virus is transmitted by bites (saliva) from a rabid animal; and hantaviruses are spread from rodents to humans by aerosols in dust from rodent excreta [19,20]. Zoonotic agents such as *Salmonella* and *Leptospira* spp. can spread from wildlife to humans indirectly through contaminated food and water [21]. Thus, it is important that Australian wildlife veterinarians are equipped with sufficient skills to protect themselves and their communities from potential wildlife zoonoses and emerging infectious diseases. The findings from this survey call for an urgent need to ensure graduate competency in diagnosing zoonoses in wildlife.

The limitations of this study are those typical to online surveys, which include the unreliability of email lists and the unwillingness of some students to participate [22]. Given that the survey was voluntary, it cannot be guaranteed that the overall responses were representative of all veterinary students in Australia. While every effort was made to encourage student participation, the overall average response rate was 21.8%. Although this is somewhat standard for surveys of this nature, the students who did respond may have been particularly committed to One Health and hence more likely to participate in the survey. Thus, it could be argued that they may not necessarily be representative of the general veterinary student body at each institution. In general, females were more represented than males. Nonetheless, the gender composition of 68% (Table 2) female in this cohort is similar to the gender composition of new veterinary graduates in Australia (approximately 80% female) [23].

## 5. Conclusions

This study is only one of several that need to be conducted to gauge the perceptions and expectations of future veterinarians towards their anticipated contributions to One Health and zoonosis management. Training efforts are required to reassure future veterinarians on aspects of the diagnosis of zoonotic diseases across all species. In relation to Australian veterinarian students’ attitudes to being involved in One Health, they are willing to take on the role of educating the public and assisting physicians when it comes to zoonoses. In the present survey, despite being pilot in nature and having a limited sample size, all respondents were willing to assist physicians in the management of zoonotic cases involving both patients and clients.

## Figures and Tables

**Table 1:** Survey questions and statements.

Topics/Subtopics	
Demographic Characteristics:	
1	State your age
2	Select your gender
3	Select the university with which you are affiliated
Beliefs and Attitudes (Rated on a 5-Point Likert Scale):	
4	One Health is an important approach that will shape the veterinary/animal health profession
5	As a veterinarian, it is my duty to promote the One Health approach
6	There are enough practical frameworks for veterinarians to follow or promote issues about One Health
7	It is important that my contribution to control/treat zoonoses will bring about a good outcome for my patient (the animal)
8	It is important that my contribution to control/treat zoonoses will bring about a good outcome for my client (the human)
9	I have a good understanding of antimicrobial stewardship guidelines for veterinarians
10	I have a good understanding of protecting myself and my staff (personal biosecurity) from potential zoonotic diseases in the workplace
Animal–Human Interprofessional Collaboration (Rated on a 5-Point Likert Scale):	
11	Veterinarians are better equipped and more knowledgeable than physicians in understanding and approaching zoonotic cases
12	Having a human health referral system to consult veterinarians’ animal-related knowledge will bring about positive changes to human health
13	I am willing to collaborate with physicians to manage zoonotic cases that affect both my animal patients and human clients
14	I am willing to collaborate with physicians to manage zoonotic cases that affect the human client only (e.g., provide advice for a client who contracted *Salmonella* infection from handling a pet reptile)
15	I am knowledgeable enough to provide advice to human clients about preventing zoonotic diseases commonly transmitted from companion animals
16	A veterinarian should always ask clients if there are any immune-compromised and immune-deficient members living with the animal as part of the basic information
17	A veterinarian should always ask clients if there are any pregnant, young or elderly members living with the animal as part of the basic information
18	It is an important part of my future work to provide advice to clients about preventing zoonotic diseases commonly transmitted from companion animals
Self-Efficacy in the Following Situations (Rated on a 5-Point Likert Scale):	
19	Your ability to diagnose common companion animal zoonotic diseases during consultation
20	Your ability to diagnose common production animal zoonotic diseases during consultation
21	Your ability to diagnose common wildlife animal zoonotic diseases during consultation

**Table 2:** Demographic characteristics of the 175 senior veterinary student survey respondents.

Characteristics	N (%)
Sex	
Male	56 (32%)
Female	119 (68%)
Age	
≤18	0
19–24	88 (50%)
25–29	63 (36%)
30–34	18 (10%)
35–39	7 (4%)
≥40	0
Student Seniority	
Final-year students	77 (44%)
Other-year students	98 (56%)
Veterinary School	
Murdoch University	91 (52%)
James Cook University	35 (20%)
University of Queensland	26 (15%)
University of Sydney	15 (8.5%)
University of Melbourne	8 (4.5)
